# Comparison of Diagnostic Performance of Commercially Available Serological and Molecular Tests for Detection of *Orientia tsutsugamushi* in South Korea: A Single-Center Prospective Study

**DOI:** 10.3390/jcm15031085

**Published:** 2026-01-29

**Authors:** Seulki Kim, Myoung Gyu Kim, Juho Jang, Jinkwan Lee, Namheon Kim, Yeji Yu, A Reum Kim, Seungjin Lim, Yong Shin, Moonsuk Bae

**Affiliations:** 1 Division of Infectious Diseases, Department of Internal Medicine, Pusan National University Yangsan Hospital, 20 Geumo-ro, Mulgeum-eup, Yangsan 50612, Republic of Korea; 2 Research Institute for Convergence of Biomedical Science and Technology, Pusan National University Yangsan Hospital, Yangsan 50612, Republic of Korea; 3 Department of Biotechnology, College of Life Science and Biotechnology, Yonsei University, 50 Yonsei-ro, Seodaemun-gu, Seoul 03722, Republic of Korea; 4 INFUSION TECH, 38 Heungan-daero, 427 Beon-gil, Anyang-si 14059, Republic of Korea; 5 Department of Internal Medicine, Pusan National University School of Medicine, Busan 46241, Republic of Korea

**Keywords:** scrub typhus, immunofluorescence assay, *Orientia tsutsugamushi*, real-time polymerase chain reaction, multiplex real-time polymerase chain reaction, immunochromatography test

## Abstract

**Background**: Scrub typhus is commonly misdiagnosed because of nonspecific clinical features and limited data on the performance of diagnostic tests. This study aimed to evaluate the accuracy of commercially available serological and molecular assays for diagnosing scrub typhus. **Methods**: Adult patients with suspected scrub typhus who visited a tertiary-care hospital in South Korea from July 2022 to December 2024 were prospectively enrolled. Scrub typhus was confirmed by either a positive real-time polymerase chain reaction (PCR) result for *Orientia tsutsugamushi* or a ≥ four-fold increase in the *O. tsutsugamushi*-specific total immunoglobulin (Ig) antibody titer on an immunofluorescence assay (IFA). The diagnostic performances of the serial IFA, an immunochromatography-based rapid diagnostic test (ICT), and multiplex real-time PCR targeting the *groEL* and *47-kDa* genes were compared. **Results**: Among 159 patients, 81 had scrub typhus and 78 did not. The sensitivity and specificity were 64% and 100% for the serial IFA, 75% and 91% for the ICT, and 95% and 100% for multiplex PCR, respectively. The area under the curve was significantly higher for the ICT (0.819) than for the acute-phase IFA (0.743, *p* = 0.02). **Conclusions**: Multiplex real-time PCR provided rapid and highly accurate confirmation of scrub typhus, and an acute-phase ICT may be an alternative to a single acute-phase IFA for early clinical decision-making.

## 1. Introduction

Scrub typhus is an acute febrile illness transmitted through the bite of the larvae of trombiculid mites; it usually manifests as headache, myalgia, rash, eschar at the bite site, generalized lymphadenopathy, or altered sensorium [1]. It is caused by *Orientia tsutsugamushi*, a Gram-negative, obligate intracellular bacterium, and represents a major public health problem worldwide, but particularly in several Asian countries [2]. As the symptoms and signs of scrub typhus are non-specific, it often resembles other acute febrile illnesses such as dengue fever, malaria, chikungunya, and leptospirosis [3]. In addition, other tick-borne infections, including severe fever with thrombocytopenia syndrome, anaplasmosis, and Q fever, may present with overlapping clinical features, further complicating the differential diagnosis [4,5]. As these conditions differ substantially in treatment strategies, disease course, and prognosis, rapid and accurate diagnosis of scrub typhus is essential for appropriate clinical management.

Serological assays are widely used and are considered the mainstay of diagnosis. The immunofluorescence assay (IFA) for detection of *O. tsutsugamushi* antibodies is considered the standard serological test [6,7]. However, it has several drawbacks, such as a wide variation in cutoff titers, lag time between infection and antibody formation, and an inability to discern between current and past infections [1,8]. These limitations can be partly overcome by determining the antibody response of two sequential samples (in the acute and convalescent phases) [1,6], although the retrospective nature of this approach remains a limitation. In addition to IFA, immunochromatographic tests have been developed to provide simpler and more rapid diagnosis [7], particularly in resource-limited settings; however, their diagnostic accuracy varies considerably across commercially available kits [9].

More recently, polymerase chain reaction (PCR)-based assays targeting *O. tsutsugamushi* have been introduced to facilitate early diagnosis, especially during the acute phase before seroconversion occurs [5]. While molecular diagnostics offer important advantages in terms of timeliness and specificity, their clinical utility depends heavily on assay design, target gene selection, specimen type, and analytical performance [6,10]. Notably, only a limited number of commercially available PCR-based diagnostic kits have demonstrated sufficient reliability and robustness for routine clinical use, and comparative evaluations of these assays using well-characterized clinical samples remain scarce [11,12,13]. Consequently, clinicians face uncertainty regarding the optimal selection and interpretation of available serological and molecular tests in real-world practice.

Therefore, this study aimed to comprehensively evaluate the diagnostic performance of commercially available serological and molecular assays for scrub typhus using clinical samples from adult patients, and to clarify the complementary roles of each diagnostic modality in clinical decision-making

## 2. Materials and Methods

### 2.1. Study Patients and Data Collection

This cross-sectional diagnostic study was conducted at Pusan National University Yangsan Hospital between July 2022 and December 2024. We prospectively enrolled all adult patients presenting with acute undifferentiated fever (≥38 °C) accompanied by one or more symptoms or signs suggestive of scrub typhus, including skin rash, eschar, headache, thrombocytopenia, elevated liver enzyme levels, lymphadenopathy, hepatosplenomegaly, or pleural effusion. A diagnosis of scrub typhus was made when at least one of two conditions was met: (1) positive results obtained via real-time PCR targeting *O. tsutsugamushi*, or (2) seroconversion or a more than four-fold increase in the *O. tsutsugamushi*-specific antibody titer upon IFA testing between serial samples collected during the disease course. Patients meeting neither criterion were assigned to the non-scrub typhus group. Data collected included the following information to identify the etiology of the fever: demographics, underlying comorbidities, presenting symptoms and physical findings, results of microbiological and radiologic evaluations, details of antimicrobial treatment (type and duration), and clinical outcomes. All enrolled patients underwent a systematic diagnostic work-up based on clinical suspicion and local epidemiology, including assessment of imaging findings, broad microbiological investigations (including blood and/or urine cultures when clinically indicated), and targeted pathogen testing (rapid antigen tests for malaria, dengue virus, influenza virus, and severe acute respiratory syndrome coronavirus 2; PCR assays for severe fever with thrombocytopenia syndrome virus and *Anaplsma phagocytophilum*; and serological testing for Epstein–Barr virus, hantavirus, human immunodeficiency virus, *Coxiella burnetii*, *Leptospira* species, and *Mycoplasma pneumoniae*). Blood was sampled from all enrolled patients during the acute and convalescent phases. Blood samples for real-time PCR were obtained exclusively during the acute phase, whereas samples for IFA were collected two to four times between the acute and convalescent phases. Patients who did not have blood samples collected during either the acute or convalescent phase were excluded from the analysis. This study was conducted in accordance with the Declaration of Helsinki, and the study protocol was approved by the Institutional Review Board of Pusan National University Yangsan Hospital (approval number: 04-2022-028) on 11 July 2022. Written informed consent was obtained from all participants.

### 2.2. Serological Testing

Serological tests for scrub typhus were conducted using commercially available IFA and immunochromatography-based rapid diagnostic test (ICT). Serum specimens for IFA were analyzed at the Green Cross Reference Laboratory (Republic of Korea). For this assay, patient sera were initially diluted at 1:40 and subsequently subjected to two-fold serial dilutions. The samples were reacted with a pooled antigen preparation of *Orientia tsutsugamushi* strains (Boryong, Gilliam, and Karp) to detect total immunoglobulin (IgM/IgG/IgA) [1,8]. The cutoff value for a single IFA-positive result for scrub typhus was defined as a total immunoglobulin (Ig) titer ≥1:40, according to the manufacturer’s recommendation. A positive serial IFA result was defined as either seroconversion or a more than four-fold increase in serial total Ig antibody titers. The ICT was performed in the hospital laboratory using a commercially available assay (Bioline Tsutsugamushi; Abbott Molecular Inc., Des Plaines, IL, USA). This test qualitatively detects total Ig antibodies (IgM/IgG/IgA) against *O. tsutsugamushi* antigens in serum samples, following the manufacturer’s protocol [1].

### 2.3. Molecular Testing

To detect *O. tsutsugamushi*, DNA was extracted from the blood of patients with suspected scrub typhus as previously described [13]. Approximately 3 mL of blood was collected into ethylenediaminetetraacetic acid (EDTA) tubes and centrifuged at 1900 *g* for 5 min. Following centrifugation, the plasma layer and buffy coat fraction were carefully transferred to sterile tubes and stored at −80 °C until analysis. DNA extraction was performed using approximately 100 μL of the combined plasma and buffy coat fraction with the I-PULL device (INFUSION TECH, Anyang-si, Republic of Korea) in four steps: (1) The plasma–buffy coat mixture was combined in the I-PULL container with 1 mL of lysis buffer (100 mM Tris-HCl [pH 8.0], 10 mM EDTA, 1% sodium dodecyl sulfate, and 10% Triton X-100), 200 µL of dimethyl sulfate (25 mg/mL), and 200 µL of amine-functionalized diatomaceous earth (ADE, 40 mg/mL); all reagents were purchased from Sigma-Aldrich (St. Louis, MO, USA). (2) The container was then sealed and incubated for 10 min, after which pressure was applied by pulling the device handle downward. (3) The polytetrafluoroethylene (PTFE) filter was rinsed with 2 mL of phosphate-buffered saline. (4) The PTFE filter was removed from the I-PULL device and bound nucleic acids were eluted from the ADE using elution buffer. Extracted DNA was stored at −20 °C until PCR analysis.

Real-time PCR was conducted using the Primera Scrub Typhus Real-Time PCR Detection Kit (INFUSION TECH, Anyang-si, Republic of Korea), which targets the *groEL* and *47-kDa* genes, according to the manufacturer’s instructions. Each reaction mixture contained 5 µL of extracted DNA and was run on a CFX96 Touch Real-Time PCR Detection System (Bio-Rad, Hercules, CA, USA). Positive and negative controls were included in each run. The PCR cycling conditions were as follows: an initial denaturation step at 95 °C for 2 min, followed by 45 cycles at 95 °C for 30 s and 61 °C for 30 s. Results were automatically interpreted by the instrument software according to pre-defined threshold and cutoff values. All tests were performed in triplicate for each sample and repeated twice in cases of discrepant results. The sample was ultimately considered positive if at least one of the triplicates was positive on the second occasion.

### 2.4. Statistical Analysis

The clinical and laboratory test results were compared between patients with scrub typhus and those without. Continuous data are described as medians and quartile ranges (IQRs) and compared between groups by using the Mann–Whitney U test. Categorical variables were assessed using Fisher’s exact test. All statistical tests were conducted as two-sided analyses, and a *p* value of <0.05 was considered indicative of statistical significance. Statistical analyses were performed using IBM SPSS Statistics for Windows (version 23.0; IBM Corp., Armonk, NY, USA). Diagnostic performance was evaluated for each test for scrub typhus, and the sensitivity of multiplex real-time PCR was additionally compared between patients who had received antibiotics before the PCR test and those who had not. The sensitivity, specificity, positive predictive value, negative predictive value, and area under the receiver operating characteristic (ROC) curve (AUC) with 95% confidence intervals (CIs) were calculated using MedCalc software (version 23.0; MedCalc Software Ltd., Ostend, Belgium).

## 3. Results

### 3.1. Patient Characteristics

A total of 159 patients with suspected scrub typhus were included in the analysis, of whom 81 were confirmed to have scrub typhus (scrub typhus group) and 78 were classified as not having scrub typhus (non-scrub typhus group). Patients in the non-scrub typhus group had bacterial infections (*n* = 20, 26%), viral infections (*n* = 7, 9%), rheumatologic diseases (*n* = 10, 13%), lymphoma (*n* = 3, 4%), Guillain–Barré syndrome (*n* = 1, 1%), or no definitive diagnosis warranted by microbiological test results (*n* = 37, 47%), as listed in Table S1. Table 1 summarized the demographic characteristics, clinical manifestations, and laboratory findings of patients with scrub typhus and those with non-scrub typhus. The median interval between symptom onset and hospital presentation was 6 days (IQR, 4–10) and did not differ significantly between patients with scrub typhus and those without (7 [4–9] days vs. 6 [3–10] days, *p* = 0.47). Eschar and skin rash were observed significantly more frequently in the scrub typhus group than in the non-scrub typhus group (80% vs. 10% and 75% vs. 23%, respectively; both *p* < 0.001). In addition, a history of outdoor exposure within three weeks prior to symptom onset was more common among patients with scrub typhus (77% vs. 32%, *p* < 0.001), and elevated liver enzyme levels were also more frequently observed in this group (68% vs. 37%, *p* < 0.001).

Of the 81 patients with scrub typhus, 54 (67%) had to be admitted, including two patients who needed to be admitted to the intensive care unit. The median time from admission to discharge was 8 (IQR, 6–9) days for the hospitalized patients. The median follow-up time for patients with scrub typhus was 14 (10–17) days. Most patients with scrub typhus (*n* = 72, 89%) were treated with doxycycline. Among the remaining patients, azithromycin (or clarithromycin) was administered in three patients (4%) due to gastrointestinal problems or contraindications such as pregnancy, and combination therapy with doxycycline and azithromycin (or clarithromycin) was provided for six patients (7%). All but one of the patients with scrub typhus recovered without sequelae.

### 3.2. Diagnostic Performance of Serological and Molecular Tests for Scrub Typhus

Seroconversion, or a more than four-fold increase in serial antibody titers (according to the IFA) was observed in 52 patients. Real-time PCR detected *O. tsutsugamushi* in 77 of the patients. Among the 81 patients, 48 (59%) had positive results for both the ≥ four-fold rise in serial IFA and the PCR test, 4 (5%) had positive results for only the ≥ four-fold rise in serial IFA, and 29 (36%) had positive results for only the PCR test. The performance of the serological and molecular tests is presented in Table 2.

The sensitivity and specificity of the ≥four-fold rise in serial IFA were 64% (95% CI, 53–75%) and 100% (95–100%), respectively (Table 2). The median duration from illness onset to the acute-phase sampling (the first sampling for the IFA) was 6 (IQR, 3–8) days, with no significant difference between the patients with positive serial IFA results and the patient with negative serial IFA results (6 [4–7] days vs. 6 [3–10] days, *p* = 0.41). The median interval between the first sampling and the last sampling for IFA was 9 (6–13) days, with no significant difference between the patients with positive serial IFA results and the patient with negative serial IFA results (9 [6–13] days vs. 9 [6–13] days, *p* = 0.92). The time point for the more than four-fold increase in the total Ig titer varied widely between patients (Figure S1a). Of fifty-two patients, thirty-eight (73%) had a more than four-fold increase in total Ig in the second week after symptoms onset, nine (17%) in the first week, and five (10%) in the third week. In patients without a ≥ four-fold increase in serial IFA titers, acute-phase total Ig levels exhibited broad variability, often beginning from relatively high baseline titers (Figure S1b). The proportion of patients with a total Ig titer ≤ 1:80 in the acute phase was significantly higher among those with a more than four-fold increase in the serial IFA than that among patients without such a change (87% vs. 55%, *p* = 0.002). Other clinical characteristics were not significantly different between the two groups. Figure S2 demonstrates the total Ig titer against *O. tsutsugamushi* in the acute-phase blood samples collected from 81 patients with scrub typhus. The sensitivity and specificity of the acute-phase single total Ig IFA with a cutoff value of ≥1:40 were 54% (95% CI, 43–65%) and 94% (86–98%), respectively.

**Figure 1:**
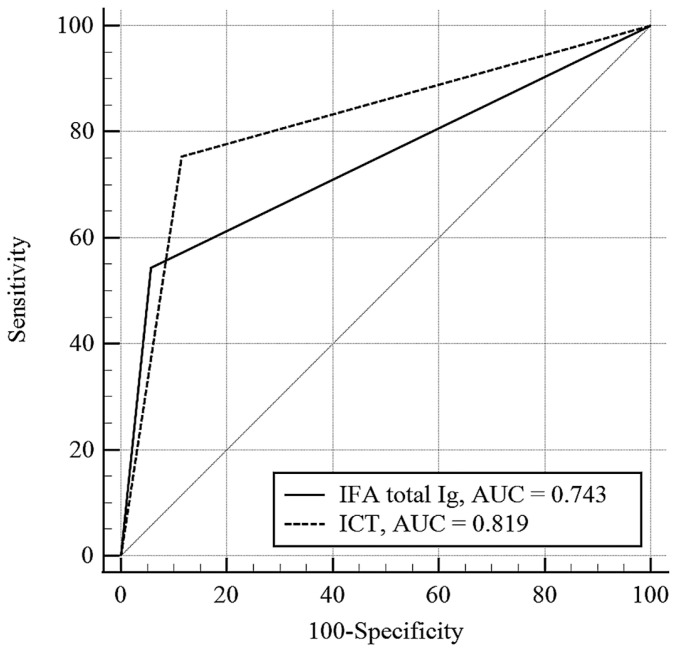
Comparison of diagnostic accuracy between ICT and IFA total Ig for acute-phase detection. Receiver operating characteristic curves represent the diagnostic performance of an immunochromatography-based rapid diagnostic test (ICT) and an acute-phase single total immunoglobulin (Ig) immunofluorescence assay (IFA) with a cutoff of ≥1:40. The area under the curve (AUC) values for ICT and IFA total Ig with a cutoff of ≥1:40 were 0.819 [95% CI, 0.747–0.877] and 0.743 [95% CI, 0.665–0.810], respectively.

The ICT was performed in 81 patients in the scrub typhus group and 69 patients in the non-scrub typhus group. The sensitivity and specificity of the ICT were 75% (95% CI, 65–84%) and 91% (82–97%), respectively. Among patients with scrub typhus, the median interval from illness onset to ICT testing was 6 (IQR, 3–8) days. The AUC of the ICT was significantly higher than that of the acute-phase single total Ig IFA at a cutoff of ≥1:40 (0.819 [95% CI, 0.747–0.877] vs. 0.743 [95% CI, 0.665–0.810], *p* = 0.02) (Figure 1).

The sensitivity and specificity of the Primera Scrub Typhus Real-Time PCR assay targeting the *groEL* and *47-kDa* genes were 95% (88–99%) and 100% (95–100%), respectively. For the Primera Scrub Typhus Real-Time PCR assay, a sample was interpreted as positive when amplification was detected for at least one of the two target genes. Of 77 patients that were Primera real-time PCR-positive, seventy-three were positive for both target genes, one for only the *groEL* gene, and three for only the *47-kDa* gene. The sensitivities for the *groEL* and *47-kDa* genes were 91% (95% CI, 83–97%) and 94% (86–98%), respectively. The sensitivity of the real-time PCR tests was compared between patients who had used antibiotics before the PCR test and those who had not. For the 62 patients who had not used antibiotics before the PCR test, the sensitivity was 95% (95% CI, 87–100%). For the 19 patients with prior use of antibiotics, the sensitivity was 95% (74–100%). The median duration of the use of antibiotics before the PCR test was 2 (IQR, 1–3) days.

## 4. Discussion

This study provides a comprehensive understanding of the performance of various diagnostic tests for scrub typhus in real-world clinical practice. The serial IFA test, traditionally regarded as the reference standard, demonstrated excellent specificity (100%) but limited sensitivity (64%). The ICT, as a point-of-care test, exhibited tolerable sensitivity (75%) and specificity (91%). The multiplex real-time PCR targeting the *groEL* and *47-kDa* genes yielded the best results, with good sensitivity and excellent specificity (95% and 100%, respectively).

A more than four-fold increase in antibody titers measured via an IFA has been regarded as the gold standard for diagnosis of scrub typhus [6]. However, its sensitivity in this study was only 64%. This reduced sensitivity may be explained by the discrepancy between the timing of clinical recovery and the timing of dynamic changes in antibody titers. Most patients with scrub typhus visited the hospital within 1 week of the onset of symptoms (median, 6 days), and they generally recovered within 2 weeks of illness onset (median admission time, 8 days). More than half of the patients with positive IFA results (73%) exhibited a more than four-fold increase in the serial antibody titers within 2 weeks of symptom onset, whereas 17% exhibited such changes earlier and 10% exhibited such changes later. In addition, antibody titers demonstrated substantial interindividual variability even among patients presenting at similar stages of illness. These findings suggest that the variability in the kinetics of humoral responses during recovery may limit the sensitivity of relying solely on a more than four-fold increase in antibody titers as a diagnostic criterion.

In a recent meta-analysis, the diagnostic performance of total antibody ICTs varied widely [9], with sensitivities ranging from 20.9% to 99.1% and specificities from 67.9% to 100.0%. Considerable heterogeneity was observed even among studies of products from the same manufacturer, which may partly reflect differences in the reference standards used across studies. Many studies relied on a single acute-phase IFA or enzyme-linked immunosorbent assay titer as the reference comparator, despite the lack of consensus on optimal positivity cutoffs. In contrast, studies employing more standardized reference methods, such as PCR confirmation and comparable timing of acute-phase sample collection, demonstrated more consistent diagnostic performance. For example, the sensitivity and specificity ranges of the SD Bioline Tsutsugamushi test were consistent, at 66.7–73.2% and 89.5–98.4%, respectively [8,14]. Although we evaluated a different ICT kit in this study (Bioline Tsutsugamushi; Abbott Molecular Inc.), the observed sensitivity (75%) and specificity (91%) were comparable to those reported in the previous studies. Notably, when applying the manufacturer-recommended cutoff of ≥1:40 for the total antibody IFA, its diagnostic performance in acute-phase samples was lower than that of the ICT (AUC of IFA = 0.743 vs. AUC of ICT = 0.819, *p* = 0.02). Given the additional advantages of the ICT, including lower cost, operational simplicity, and rapid turnaround time, this finding further supports its clinical utility for early diagnosis and clinical decision-making.

Several real-time PCR assays targeting the *47-kDa*, *groEL*, *56-kDa*, or *16S rRNA* genes have been reported. The sensitivity of real-time PCR among patients with scrub typhus ranges from 70.6% to 91.9% [11,12,15,16,17], depending on the study site and the reference test used to define a positive result. Moreover, most of the real-time PCR assays encounter the problem of false-negative results because of genetic polymorphisms of *O. tsutsugamushi* and the level of bacteremia, which is affected by the duration of the illness, disease severity, and antibiotic administration [12,17,18,19]. The multiplex real-time PCR assay targeting the *groEL* and *47-kDa* genes may overcome these problems [13]. It yielded good sensitivity (95%) and excellent specificity (100%) even as a single diagnostic test. This assay also exhibited no significant changes in sensitivity depending on whether antibiotics were used before PCR testing (95% with prior use vs. 95% without prior use). In addition, because it is used to simultaneously detect two genes specific to *O. tsutsugamushi*, the risk of false negatives is low, as the probability of polymorphism in both genes is low.

Although molecular tests, especially real-time PCR, have been proposed as the new gold standard tests for the diagnosis of scrub typhus, the serological tests still have certain advantages. Of 81 patients with scrub typhus, four (5%) had positive results only for the serial IFA, with negative results for the real-time PCR test. This disagreement between test results may be due to the serological diversity and genetic polymorphisms of *O. tsutsugamushi* [20,21,22]. Few studies have been reported on the serotype distribution and genetic/phylogenetic information of *O. tsutsugamushi* in the Republic of Korea [23,24]; thus, further research is needed on this subject.

This study has some limitations. First, the number of enrolled patients was relatively small, which may have limited the statistical power to fully assess the diagnostic performance of each test. Second, because the study comprised patients who visited a tertiary-care hospital, patients presented with mild disease may not have been included. Third, as this study was conducted under real-world clinical conditions, the follow-up duration was relatively short, generally less than one month.

## 5. Conclusions

In this real-world clinical evaluation of commercially available diagnostic assays for scrub typhus, multiplex real-time PCR targeting the *groEL* and *47-kDa* genes provided the most rapid and accurate laboratory confirmation during the acute phase, outperforming serological testing based solely on dynamic antibody responses. Acute-phase ICTs showed acceptable accuracy and served as a practical alternative to a single acute-phase IFA for immediate clinical decision-making. Although serial IFA testing is constrained by delayed antibody kinetics, it remains complementary by increasing diagnostic sensitivity and supporting diagnostic confidence when molecular results are negative. Overall, our findings support an integrated diagnostic strategy combining molecular and serological methods to optimize timely and accurate diagnosis of scrub typhus in routine clinical practice.

## Figures and Tables

**Table 1:** Clinical characteristics of 81 patients with scrub typhus and 78 patients with non-scrub typhus.

	Total (*n* = 159)	Scrub Typhus (*n* = 81)	Non-Scrub Typhus (*n* = 78)	*p* * Value
Age, median (IQR), year	67 (57–74)	67 (58–73)	67 (57–77)	0.88
Male sex	76 (48)	37 (46)	39 (50)	0.59
Exposure to field	87 (55)	62 (77)	25 (32)	<0.001
Underlying disease ^†^	60 (38)	27 (33)	33 (42)	0.24
Clinical symptoms				
Eschar	73 (46)	65 (80)	8 (10)	<0.001
Skin rash	79 (50)	61 (75)	18 (23)	<0.001
Headache	37 (23)	19 (23)	18 (23)	0.96
Lymphadenopathy	49 (31)	21 (26)	28 (36)	0.17
Hepatomegaly	13 (8)	7 (9)	6 (8)	0.83
Splenomegaly	23 (14)	11 (14)	12 (15)	0.75
Pleural effusion	54 (34)	21 (26)	33 (42)	0.029
Laboratory findings ^‡^				
Thrombocytopenia	66 (42)	39 (48)	27 (35)	0.083
Increased liver enzyme	84 (53)	55 (68)	29 (37)	<0.001

Data are presented as number (%) of patients unless otherwise indicated. Abbreviations: IQR, interquartile range. * Group comparisons were performed using the Mann–Whitney U test or Fisher’s exact test, as appropriate. A two-sided *p* value < 0.05 was considered statistically significant. ^†^ Underlying disease included diabetes mellitus, chronic respiratory disease, chronic kidney failure, congestive heart failure, liver cirrhosis, malignancy, and receiving immunosuppressive treatment. ^‡^ Thrombocytopenia was defined as a platelet count < 140,000/mm^3^. Increased liver enzyme levels were defined as a serum AST or ALT > 1.5-fold the upper limit of normal.

**Table 2:** Comparison of Diagnostic performance of serological and molecular tests for scrub typhus.

Diagnostic Test *	Sensitivity (95% CI)	Specificity (95% CI)	PPV (95% CI)	NPV (95% CI)	Accuracy (95% CI)
Serological tests					
≥4-fold rise in serial IFA results ^†^	64 (53–75)	100 (95–100)	100 (93–100)	73 (67–78)	82 (75–87)
ICT results	75 (65–84)	91 (82–97)	91 (82–96)	75 (67–82)	82 (75–88)
Molecular tests					
Primera real-time PCR	95 (88–99)	100 (95–100)	100 (95–100)	95 (88–98)	98 (94–99)
*groEL* gene	91 (83–97)	100 (95–100)	100 (95–100)	92 (85–96)	96 (91–98)
*47*-*kDa* gene	94 (86–98)	100 (95–100)	100 (95–100)	94 (87–97)	97 (93–99)

Abbreviations: CI, confidence interval; ICT, immunochromatography-based rapid diagnostic test; IFA, immunofluorescence assay; NPV, negative predictive value; PCR, polymerase chain reaction; PPV, positive predictive value; Primera real-time PCR, Primera Scrub Typhus Real-Time PCR Detection Kit. * All the assays were performed on 81 patients with scrub typhus and 78 patients in the non-scrub typhus group except for ICT, for which 81 patients with scrub typhus and 69 patients in the non-scrub typhus group were included. ^†^ A positive serial IFA result was defined as either seroconversion or a more than four-fold increase in serial total immunoglobulin antibody titers.

## Data Availability

The original contributions presented in this study are included in the article/Supplementary Material. Further inquiries can be directed to the corresponding authors.

## Supplementary Materials

The following supporting information can be downloaded at: https://www.mdpi.com/article/10.3390/jcm15031085/s1, Figure S1: Distribution of total Ig titer against *Orientia tsutsugamushi* in blood samples throughout the disease course; Figure S2: Total Ig titer against *Orientia tsutsugamushi* in acute-phase blood samples collected 0–15 days from the onset of symptoms; Table S1: Non-scrub typhus diseases identified in this study.

## Abbreviations

The following abbreviations are used in this manuscript:

PCR	Polymerase chain reaction
Ig	Immunoglobulin
IFA	Immunofluorescence assay
ICT	Immunochromatography-based rapid diagnostic test
CI	Confidence interval
EDTA	Ethylenediaminetetraacetic acid
ADE	Amine-functionalized diatomaceous earth
PTFE	Polytetrafluoroethylene
Q	Quartile
AUC	Area under the receiver operating characteristic curve
ROC	Receiver operating characteristic
