# Supplementary material for: Comparison of Diagnostic Performance of Commercially Available Serological and Molecular Tests for Detection of Orientia tsutsugamushi in South Korea: A Single-Center Prospective Study
Source: J Clin Med. 2026 Jan 29;15(3):1085. doi: 10.3390/jcm15031085 (PMC12898047; doi:10.3390/jcm15031085)
Supplement: Supplementary file 1 [file jcm-15-01085-s001.zip › jcm-4112751-supplementary.pdf]

Journal of
Clinical Medicine

MDPI

Article

Comparison of Diagnostic Performance of Commercially Available Serological and Molecular Tests for Detection of *Orientia tsutsugamushi* in South Korea: A Single-Center Prospective Study

Table S1. Non-scrub typhus diseases identified in this study

Diagnosis	Number of patients
Bacterial infection	
Bacteremia	14
Urinary tract infection	3
Human granulocytic anaplasmosis	2
Tuberculosis lymphadenitis	1
Viral infection	
Severe fever with thrombocytopenia syndrome	2
Dengue fever	1
Infectious monocytosis	1
Influenza	1
Severe acute respiratory syndrome coronavirus-2 infection	1
Acute human immunodeficiency virus syndrome	1
Rheumatologic disease	
Systemic lupus erythematosus	2
Axial spondylitis	3
Adult-onset Still's disease	1
Inflammatory myositis	1
ANCA-associated vasculitis	1
Gout	1
Kikuchi disease	1
Lymphoma	3
Guillain-Barré syndrome	1
No definitive diagnosis	37
Total	78

ANCA, anti-neutrophil cytoplasm antibody

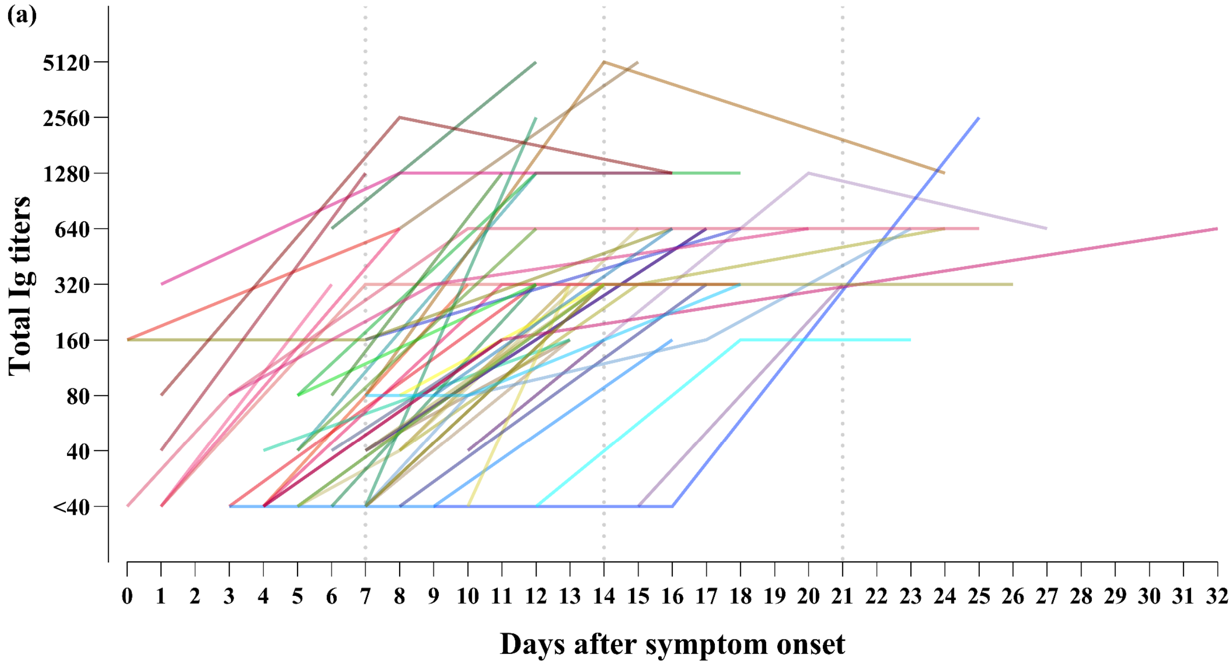

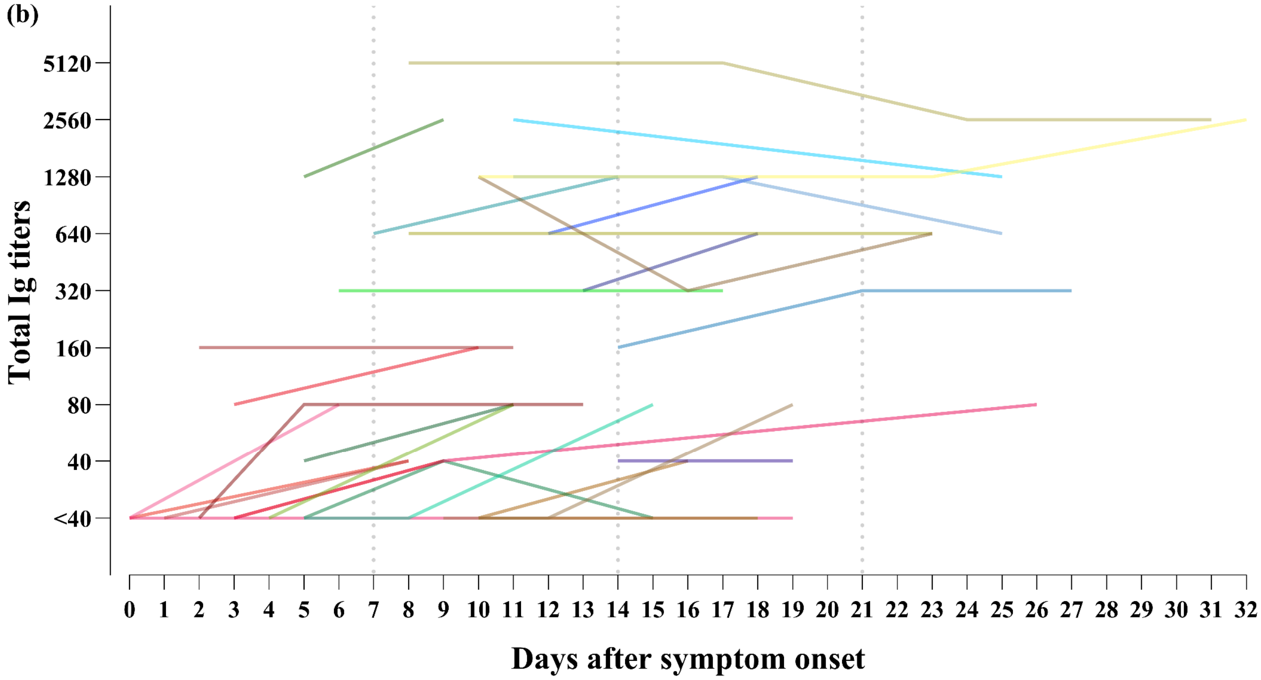

Figure S1. Distribution of total Ig titer against *Orientia tsutsugamushi* in blood samples throughout the disease course. (a) 52 patients who had a greater than four-fold increase in the serial immunofluorescence assay (IFA); and (b) 29 patients who had no significant change in the serial IFA.

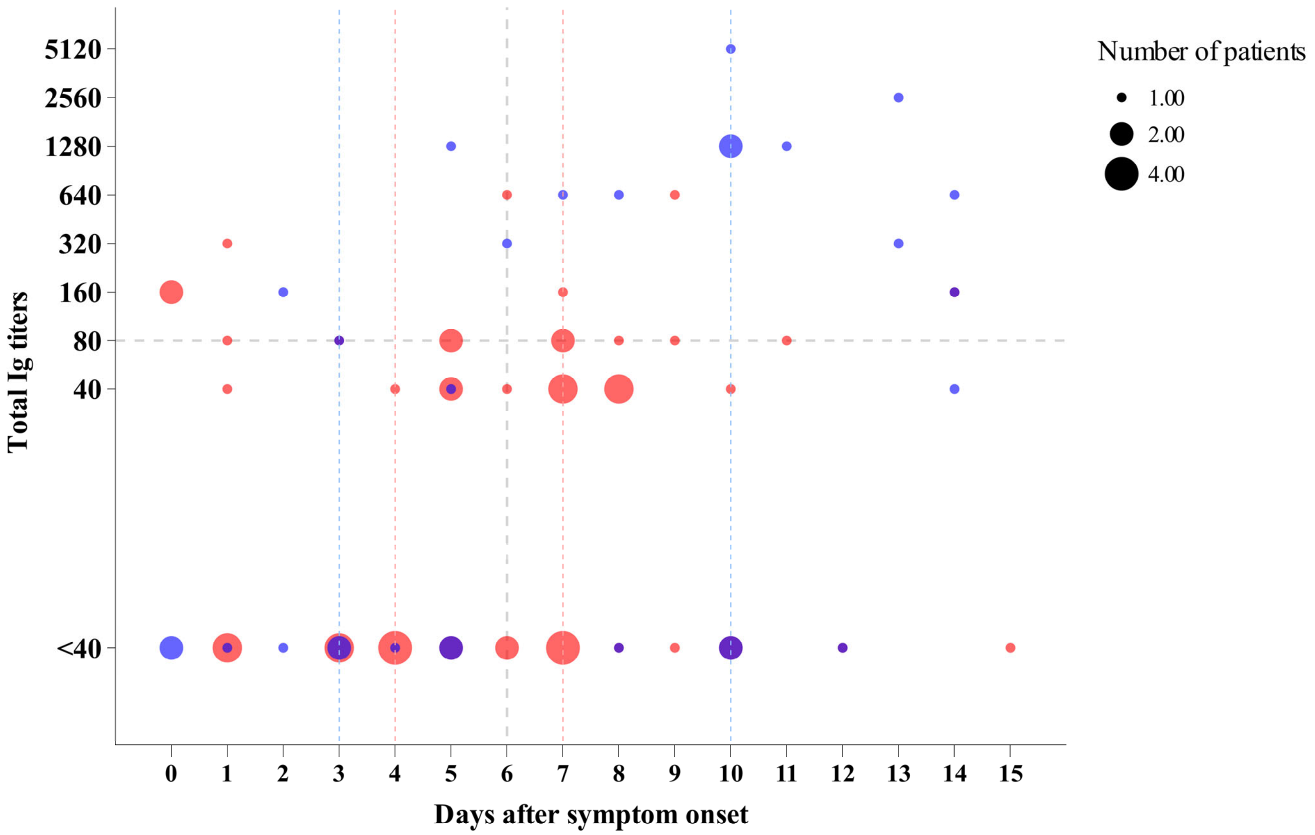

Figure S2. Total Ig titer against *Orientia tsutsugamushi* in acute phase blood samples collected 0–15 days from the onset of symptoms. Solid red dots represent patients who had a greater than four-fold increase in the serial total Ig titers via immunofluorescence assay (IFA) and solid blue dots represent those who had no significant change in the serial total Ig titers via IFA.
